# Let-7a regulates expression of β_1_-adrenoceptors and forms a negative feedback circuit with the β_1_-adrenoceptor signaling pathway in chronic ischemic heart failure

**DOI:** 10.18632/oncotarget.14436

**Published:** 2017-01-02

**Authors:** Yue Du, Mingyu Zhang, Wei Zhao, You Shu, Ming Gao, Yanan Zhuang, Ti Yang, Wei Mu, Tingting Li, Xin Li, Fei Sun, Zhenwei Pan, Yanjie Lu

**Affiliations:** ^1^ Department of Pharmacology (State-Province Key Laboratories of Biomedicine-Pharmaceutics of China, Key Laboratory of Cardiovascular Medicine Research, Ministry of Education), College of Pharmacy, Harbin Medical University, Harbin, Heilongjiang 150081, P. R. China; ^2^ Northern Translational Medicine Research and Cooperation Center, Heilongjiang Academy of Medical Sciences, Harbin, Heilongjiang 150081, P. R. China

**Keywords:** chronic ischemia heart failure, let-7a, β_1_-AR, GATA4

## Abstract

**Background:**

The aim of the present study was to investigate the role of microRNA (miRNA) let-7a in down-regulation of β_1_-adrenoceptors (β_1_-AR) and elucidate the underlying mechanism of chronic ischemia heart failure (CIHF) in rats.

**Methods and Results:**

CIHF model was established by occlusion of coronary artery for 4 weeks. β_1_-AR level was obviously down-regulated and let-7a up-regulated in the failing heart 4 weeks after myocardial infarction. Overexpression of let-7a inhibited β_1_-AR expression in neonatal rat ventricular cells (NRVCs), which was abolished by anti-let-7a antisense inhibitor. The lentivirus vector containing precursor let-7a (len-pre-let-7a) further down-regulated the reduced β_1_-AR level by CIHF and the effect was reversed by len-AMO-let-7a. Len-negative control did not produce any significant influence on β_1_-AR expression. Importantly, there exists a negative feedback loop associated with β_1_-AR regulation through β_1_-AR/cAMP/PKA/GATA4/let-7a/β_1_-AR signaling pathway in CIHF. As demonstrated, GATA4 was activated by β_1_-AR up-regulation through cAMP-PKA signaling pathway in early phase of ischemia, then GATA4 positively regulated let-7a expression which in turn suppressed β_1_-AR expression.

**Conclusions:**

Let-7a regulates β_1_-AR expression and forms a negative feedback loop with β_1_-AR signaling pathway in ischemic heart failure. This study provides a new insight into the differential expression of β_1_-AR in early and later phase of myocardial ischemia.

## INTRODUCTION

Chronic ischemic heart failure (CIHF) remains the leading cause of morbidity and mortality worldwide [1]. CIHF is a complicated pathophysiological process which involves a number of pathways and factors. Adrenergic receptors are functionally involved in numerous processes underlying both aging and cardiovascular disease, in particular heart failure [2]. β_1_-adrenoceptor (β_1_-AR) demonstrates differential expression at different stages of heart failure, showing up-regulated in early stage of heart failure and down-regulated in late stage of the disease. However, the molecular mechanism of down-regulated expression of β_1_-AR as an adaptive effect for cardioprotection in CIHF remains unclear.

The β-adrenergic receptor (β-AR) and its mediated sympathetic activities play a primary role in governing cardiac rhythm/heart rate, cardiac excitation/conduction, and cardiac contraction through regulating the associated cellular signal transductions [3]. β-ARs can be divided into three categories in heart based on their differential pharmacological properties: β_1_-AR, β_2_-AR and β_3_-AR [4]. The β_1_-AR is the main subtype for cardiac function regulation, and it acts through activating the G-protein/adenylate cyclase/protein kinase A (PKA) signaling pathway [5]. Interestingly, the expression level of β_1_-AR changes dynamically at different stages of myocardial ischemia [6], being down-regulated in CIHF but up-regulated in acute myocardial infarction [7]. GATA4 is a transcription factor that can directly regulate the expression of a large number of cardiac genes upon activation by the β_1_-AR-cAMP/PKA signaling pathway. In this way, it is critically involved in the regulation of cardiac development [8], cardiac hypertrophy and apoptosis [9], and gene mutation [10] caused by a variety of heart diseases.

MicroRNAs (miRNAs) are small single-stranded non-coding RNAs, which mostly suppress protein translation by binding to the 3’UTR of mRNAs [11]. Numerous studies have demonstrated that miRNAs are involved in the development of cardiovascular diseases, such as myocardial ischemia, arrhythmias, cardiac hypertrophy, myocardial fibrosis, etc [12, 13]. For instance, overexpression of miR-1 deleteriously affects cardiac conduction and membrane depolarization in myocardial infarction [14]. MiR-26 and miR-328 control vulnerability of atrial fibrillation [15]. Overexpression of miR-101 inhibits interstitial fibrosis of infarct hearts [16]. MiR-133a protects the heart from pressure-overload induced injury by inhibiting the expression of β_1_-Ars [17].

Let-7 belongs to a miRNA family containing 13 members sharing the same seed sequence thereby the same set of target genes [18], and is affluently expressed in the heart [19]. Studies discovered the deregulation of the let-7 members in cardiovascular diseases, such as cardiac hypertrophy, cardiac fibrosis, and myocardial infarction [20]. Our previous study revealed that let-7 is considerably down-regulated in the setting of acute myocardial ischemia which results in the upregulation of β_1_-ARs and the associated arrhythmogenesis and dysfunction of the heart. Let-7 replacement rescues cardiac function mainly through its anti-arrhythmic efficacy [21]. Intriguingly, in our preliminary studies in a rate model of chronic myocardial ischemia, members of the let-7 miRNAs family were found upregulated, whereas let-7a showed the opposite expression. The mechanisms for let-7 up-regulation and the possible pathophysiological role of let-7 upregulation in CIHF remained unknown.

The present work was conducted to elucidate the mechanism by which let-7a is up-regulated and the influence it produces to the heart in a model of CIHF. Our findings allowed us to establish a negative feedback circuit as a mechanism for abnormal up-regulation of let-7a and to verify the up-regulation of let-7a as a mechanism for the down-regulation of β_1_-ARs in heart failure.

## RESULTS

### Down-regulation of β_1_-ARs and up-regulation of let-7 in chronic ischemic failing heart

**Figure 1:**
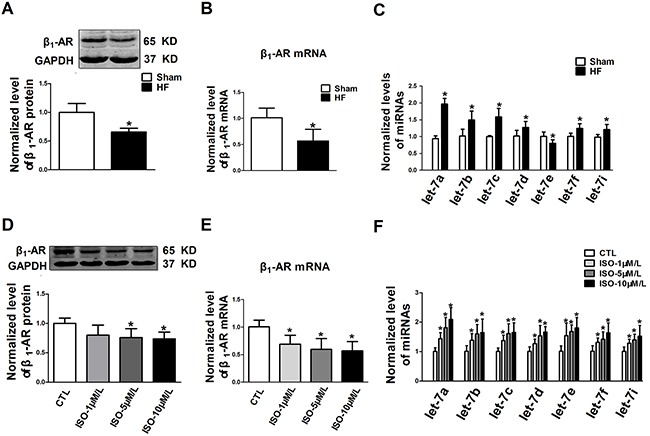
Level of β1-AR and let-7 in chronic ischemic failing hearts and ISO-treated neonatal rat ventricular cardiomyocytes (NRVCs) **A-C.** β1-AR protein, β1-AR mRNA and let-7 family levels in the hearts of ischemic heart failure rats; **D-F.** β1-AR protein, β1-AR mRNA and let-7 family levels in NRVCs treated with isoproterenol (ISO) for 72 h. HF, heart failure; Ctl, control. Data are expressed as mean ± SD; n = 5 animals or 4 batches of cells for each group; *P < 0.05 vs Sham or Ctl.

The expression level of β_1_-ARs was decreased by 34.0 ± 6.3% in failing hearts compared with those in non-failing hearts (Figure 1A). β_1_-AR mRNA was also decreased by 44.3 ± 22.2 % in failing heart tissues (Figure 1B). These results are consistent with the previous studies showing down-regulation of β_1_-ARs in chronic heart failure [7]. On the other hand, most of detected members of the let-7 family (let-7a, b, c, d, f and i) were elevated, while let-7e decreased in CIHF compared with the sham group (Figure 1C). Among which, let-7a was the most notably up-regulated miRNA. The NRVCs were treated with varying concentrations (1, 5, and 10 μM) of ISO for 72 h to produce the cellular model of down-regulation of β_1_-AR [22]. In agreement with the in vivo data, reciprocal changes of expression of β_1_-AR and let-7 miRNAs were also observed in ISO-treated NRVCs. Under such a condition, the levels of β_1_-AR protein and mRNA were both reduced in a concentration dependent manner (Figure 1D and 1E), whereas, the members of let-7 family, particularly let-7a, were markedly up-regulated (Figure 1F).

### Let-7 regulates expression of β_1_-ARs in cardiomyocytes

**Figure 2:**
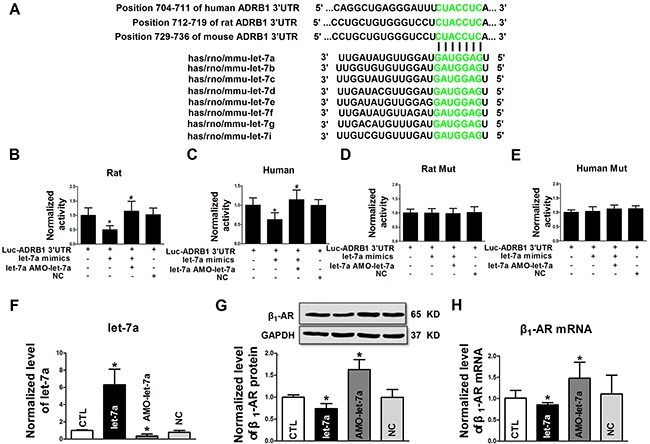
Experimental verification of β1-AR as a target of let-7a **A.** Alignment of the sequences of let-7 family (bottom) with their target sites in the 3’UTRs of human, rat and mouse β1-AR mRNAs (top). The complementary nucleotides are highlighted in green; **B** and **C.** Luciferase reporter gene activities generated by luciferase vectors carrying wild-type 3’UTRs of rat and human β1-adrenergic receptor (ADRB1), respectively; **D** and **E.** Luciferase reporter gene activities generated by luciferase vectors carrying mutant 3’UTRs of rat and human ADRB1, respectively; **F.** Let-7a levels in NRVCs transfected with let-7a mimic, AMO-let-7a or negative control (NC); **G** and **H.** β1-AR mRNA and protein levels in NRVCs transfected with let-7a, AMO-let-7a or NC group. Data are expressed as mean ± SD; n = 4-6 batches for each group; *P < 0.05 vs Ctl (no treatment); #P < 0.05 vs the group transfected with let-7a.

TargetScan miRNA database was used to predict a binding site at the 3’UTR of β_1_-AR mRNA for all members of the let-7 family, which is highly conserved among human, rat and mouse (Figure 2A). We therefore conducted a series of experiments to experimentally verify the targeting relationship between let-7a and β_1_-ARs. The first of such experiments was luciferase activity assay in HEK-293 cells. Our results displayed that luciferase activities were significantly inhibited by let-7a with wild-type 3’UTR of rat and human ADRB1, which were canceled by co-transfection of AMO-let-7a (Figure 2B and 2C). Let-7a produced no effects on luciferase activities of constructs with mutant 3’UTR of rat and human ADRB1 (Figure 2D and 2E).

The transfection efficiency of let-7a and the effectiveness of AMO-let-7a to knockdown endogenous let-7a were determined by qRT-PCR in NRVCs. Let-7a was increased by 6.3-fold by transfection of let-7a mimics and reduced by 35.6 ± 22.2 % by transfection of AMO-let-7a (Figure 2F). Consistent with luciferase assay, let-7a overexpression inhibited β_1_-ARs protein expression in NRVCs. Conversely, knockdown of endogenous let-7a by AMO-let-7a increased β_1_-AR protein level relative to the control group (Figure 2G). β_1_-AR mRNA level was similarly affected by let-7a (Figure 2H). In all cases, negative control constructs failed to affect the expression of let-7a and β_1_-ARs.

### Effects of let-7a knockdown on β_1_-ARs expression and cardiac function of rats with CIHF

**Figure 3:**
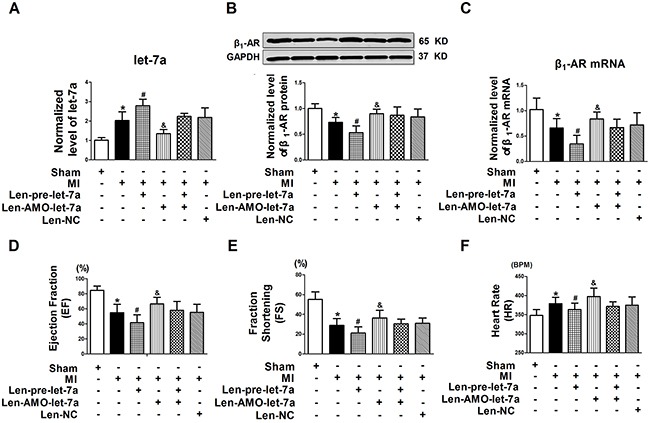
Inhibition of let-7a alleviated the downregulation of β1-AR and improved cardiac function in a rat model of CIHF **A.** let-7a in CIHF treated with len-pre-let-7a, len-AMO-let-7a, or len-NC; **B** and **C.** β1-AR protein and mRNA expression in CIHF treated with len-let-7a, len-AMO-let-7a, or len-NC; **D** and **E.** Cardiac function of rats by echocardiography; F. Heart rate (HR) of rats in models of CIHF. Date are expressed as mean ± SD, n = 5-10 in each group; *P < 0.05 vs Sham,#P < 0.05 vs MI,&P < 0.05 vs MI + Len-pre-let-7a.

The level of let-7a was significantly up-regulated by 2.0-fold in the CIHF rats than controls, and len-pre-let-7a further increased let-7a expression by 0.8-fold compared with the CIHF group. Administration of len-AMO-let-7a reduced let-7a level in CIHF rats, while Len-NC had no effect (Figure 3A). Of note, len-let-7a profoundly strengthened the CIHF-induced down-regulation of β_1_-AR protein level, which was repealed by co-treatment with len-AMO-let-7a. Moreover, len-AMO-let-7a alone reversed the CIHF-induced down-regulation of β_1_-ARs. β_1_-AR expression was not influenced by len-NC alone (Figure 3B). Similar changes in β_1_-AR mRNA level were seen (Figure 3C).

Len-pre-let-7a caused a significant deterioration of cardiac function, as reflected by decreased ejection fraction (EF) and fractional shortening (FS) in CIHF rats. Oppositely, len-AMO-let-7a enhanced EF from 41.5 ± 10.6% to 66.6 ± 8.7% (*P* < 0.05) and FS from 21.1 ± 6.3% to 36.4 ± 7.7% (*P* < 0.05) compared to the len-let-7a group (Figure 3D and 3E). And then the heart rate (HR) was also decreased by len-pre-let-7a compared with the MI group, however len-AMO-let-7a could increase the heart rate from from 379 ± 16 to 398 ± 22 Beat Per Minute (BPM) (*P* < 0.05) (Figure 3F). Reduced heart rate in let-7a-treated MI rats was likely attributed to inhibition of β_1_-AR in heart sinus.

### GATA-4 participates in the regulation of let-7a and β_1_-AR expression

GATA4 is a cardiac specific transcription factor that plays a key role in the regulation of cardiac physiology and development. Our initial computational analysis using the JASPAR database predicted that there are putative binding regions for GATA4 in the upstream regulatory domain of the let-7a gene.

**Figure 4:**
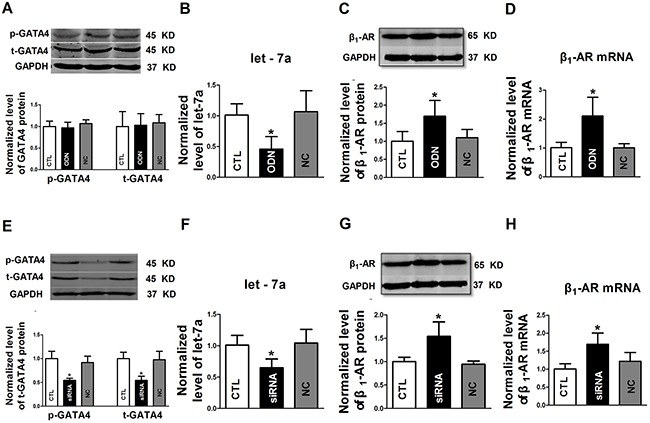
The regulatory effects of GATA4 on let-7a and β1-AR expression **A.** Effects of decoy ODN (oligodeoxynucleotide) on the expression of total GATA4 protein (t-GATA4) and phosphorylated GATA4 (p-GATA4); **B.** Effects of GATA-4 decoy ODN on let-7a expression; **C** and **D.** Effects of GATA-4 decoy ODN on β1-AR protein and mRNA levels, respectively; **E.** Effects of GATA-4 siRNA on the expression of t-GATA4 and p-GATA4; **F.** Effects of GATA-4 siRNA on let-7a expression; **G** and **H.** Effects of GATA-4 siRNA on β1-AR protein and mRNA levels, respectively. Date are expressed as mean ± SD, n = 3-6 in each group; *P < 0.05 vs Ctl (no treatment).

To explore the possible role of GATA4 in regulating let-7a transcription, the decoy ODN (oligodeoxynucleotide) for GATA4 was transfected into NRVCs. As expected, the levels of total GATA4 and the phosphorylated form of GATA4 remained unaffected by the decoy ODN (Figure 4A). However, let-7a level was significantly diminished (Figure 4B), and β_1_-AR was pronouncedly up-regulated at both protein and mRNA levels (Figure 4C and 4D). Meanwhile, GATA4 siRNA was also used to testify the role of GATA4 in regulation of let-7a and β_1_-AR in NRVCs. Unsurprisingly, the decreased levels of total GATA4 and the phosphorylated form of GATA4 were observed after pretreated with GATA4 siRNA (Figure 4E). Let-7a was decreased in GATA4 siRNA transfection group (Figure 4F), and the levels of β_1_-AR protein and mRNA increased (Figure 4G and 4H). These data suggested that GATA4 regulates β_1_-AR expression through let-7a.

### Prolonged activation of β_1_-AR and Its downstream pathway factor cAMP forms a regulation of negative feedback loop on β_1_-AR expression through GATA4

**Figure 5:**
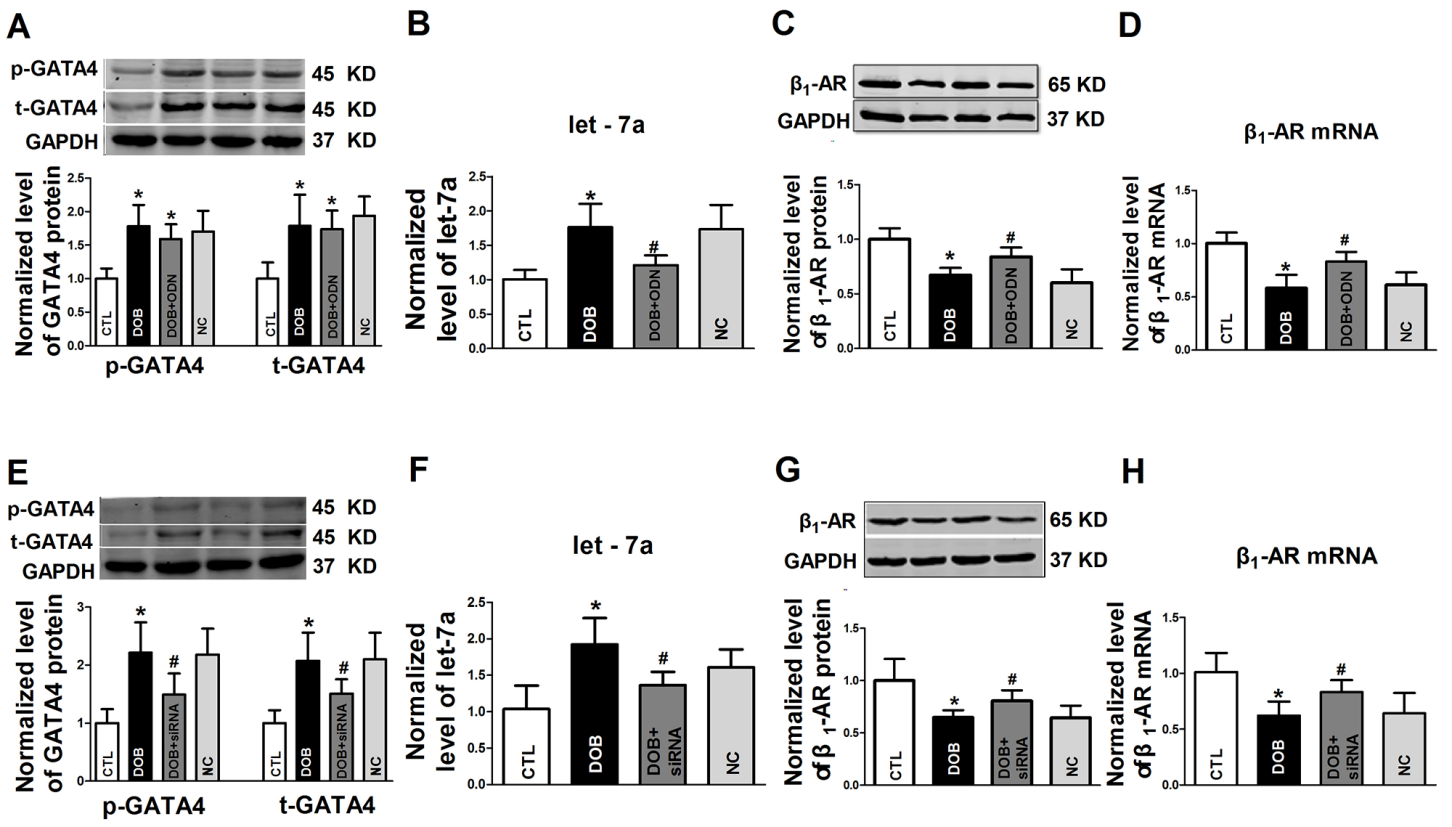
The role of GATA4 on β1-AR activation in regulation of expression of let-7a and β1-AR in NRVCs **A.** Expression of t-GATA4 and p-GATA4 in NRVCs treated with DOB or in combination with GATA-4 decoy ODN; **B-D.** Effects of GATA-4 decoy ODN on DOB regulated expression of let-7a, β1-AR protein andβ1-AR mRNA, respectively; **E.** Expression of t-GATA4 and p-GATA4 treated with dobutamine (DOB) or in combination with GATA-4 siRNA; F-H. Effects of GATA-4 siRNA on DOB regulated expression of let-7a, β1-AR protein and β1-AR mRNA, respectively. Date are expressed as mean ± SD, n = 4-5 in each group; *P < 0.05 vs Ctl (no treatment); #P < 0.05 vs pretreated DOB group.

While the above experiments provided strong evidence for the role of GATA4 in the regulation of let-7a transcription, we pretreated the NRVCs with dobutamine (DOB, a selective β_1_-AR agonist, 10 μM) for 72 h. We observed that the levels of the total protein (t-GATA4) and phosphorylated form of GATA4 (p-GATA4) were increased compared with the control groups (Figure 5A). In the presence of DOB, the level of let-7a was up-regulated, which was abolished by the decoy ODN of GATA4 (Figure 5B). The decoy ODN of GATA4 also effectively set back the expression of β_1_-AR protein and mRNA suppressed by DOB (Figure 5C and 5D). We also used the GATA4 siRNA to perform the above experiments. Both t-GATA4 and p-GATA4 were inhibited by GATA4 siRNA, which were up-regulated in the presence of DOB (Figure 5E). DOB-induced Let-7a upregulation was inhibited by transfection of GATA4 siRNA (Figure 5F). β_1_-AR protein and mRNA levels were both down-regulated with DOB treatment, which were restrained by GATA4 siRNA transfection (Figure 5G and 5H).

**Figure 6:**
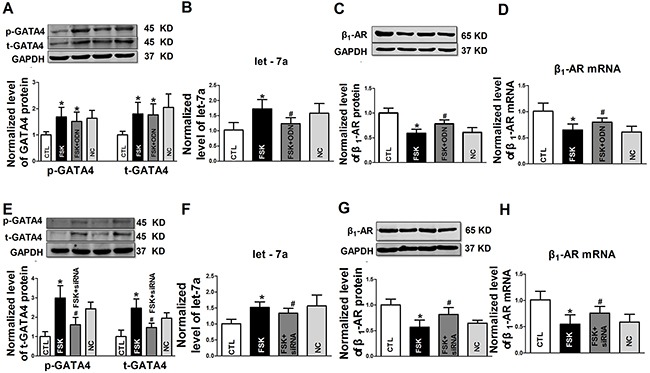
The role of GATA4 on cAMP activator forskolin in regulation in expression of let-7a and β1-AR in NRVCs **A.** Expression of t-GATA4 and p-GATA4 treated with FSK or in combination with GATA-4 decoy ODN; **B-D.** Effects of GATA-4 decoy ODN on forskolin (FSK) regulated expression of let-7a, β1-AR protein and β1-AR mRNA, respectively; E. Expression of t-GATA4 and p-GATA4 treated with FSK or in combination with GATA-4 siRNA; F-H. Effects of GATA-4 siRNA on FSK regulated expression of let-7a, β1-AR protein and β1-AR mRNA, respectively. Date are expressed as mean ± SD, n = 4-5 in each group; *P < 0.05 vs Ctl (no treatment); #P < 0.05 vs pretreated FSK group.

To verify the activation role of cAMP-PKA signaling pathway, forskolin (FSK, a cAMP activator, 10 μM) was used to treat the NRVCs for 72 h. The results showed the levels of t-GATA4) and p-GATA4 were upregulated by FSK, which were unaffected by the co-administration of GATA4 decoy ODN (Figure 6A). FSK up-regulated the level of let-7a, and the effects were abolished by GATA4 decoy ODN (Figure 6B). In comparison, β_1_-AR protein and mRNA levels were down-regulated with FSK treatment for 72 h, which were recovered by GATA4 decoy ODN treatment (Figure 6C and 6D). Similarly, GATA4 siRNA reduced the up-regulation of t-GATA4 and p-GATA4 by FSK. In all situations, NC had no effects on the expression of GATA4 (Figure 6E). Let-7a was increased by long-term treatment of FSK, and the up-regulated expression of let-7a was reversed by GATA4 siRNA transfection (Figure 6F). The down-regulation of β_1_-AR protein and mRNA pretreated with FSK could be changed with the GATA4 siRNA treatment (Figure 6G and 6H). These data indicate that cAMP-PKA signaling pathway mediates the down-regulation of β_1_-AR by targeting GATA4 and let-7a.

## DISCUSSION

**Figure 7:**
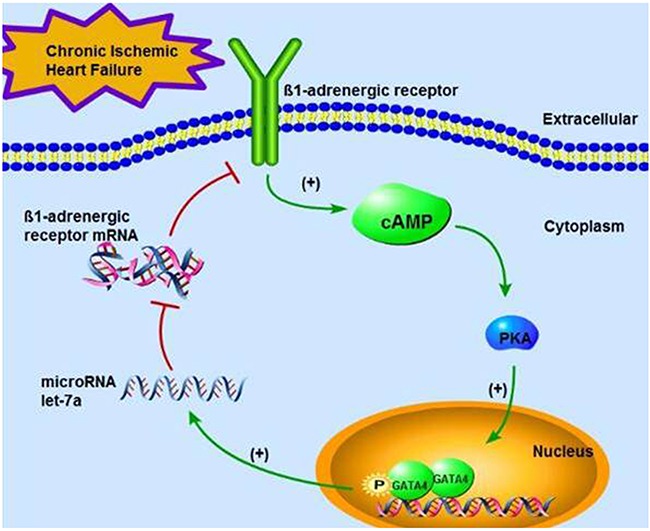
A schematic diagram illustrating the feedback circuit of the signaling pathway β1-AR—cAMP—PKA—GATA-4—let-7a—β1-AR in CIHF

It has been long recognized that β_1_-ARs are down-regulated in the setting of chronic ischemic heart failure (CIHF) [7]. The main purpose of our study was to explore the mechanisms of β_1_-AR down-regulation in CIHF and role of let-7a in regulating the β_1_-AR/cAMP/PKA/GATA-4 signaling pathway. Our results revealed the following aspects. First, the level of let-7 family members, especially let-7a, was significantly up-regulated in CIHF, along with down-regulation of β_1_-ARs. Second, let-7a directly targeted ADRB1 mRNA 3’UTR to repress β_1_-AR expression. Third, GATA4 positively regulated the transcription of let-7a. It is conceivable based on our findings that let-7 participates in the β_1_-AR/cAMP/PKA/GATA-4 signaling pathway as an upstream component and its participation forms a negative feedback loop. We therefore proposed the modified signaling pathway: β_1_-AR↑→cAMP↑→PKA↑→GATA-4↑→let-7↑→β_1_-AR↓. This can be translated into a more physiological term: in the early stage, or the acute phase, of ischemia, the enhanced sympathetic tone induces β_1_-AR activation and the subsequent signal transduction, leading to activation of GATA4 thereby the expression of let-7a. The latter then serves to tune down the signal transduction by down-regulating expression of β_1_-ARs, as occurring in CIHF. A schematic diagram illustrating the feedback circuit is presented in Figure 7.

It was well known that β-AR played a leading role in mediating cardiac function, and β_1_-AR accounts for 70% in heart and played a major role in the development of CIHF [23–25]. β_1_-AR shows up-regulation in acute phase of ischemic heart failure and down-regulation in late phase of heart failure, both changes as adaptive effects for cardiac function and cardioprotection. In the early stage of ischemic heart failure, the up-regulation of β_1_-AR is due to ischemia caused by sympathetic nerve excitability, adrenergic hyperactivity and endogenous catecholamines substance secretion. The activation of β_1_-AR could accelerate sinus rhythm, strengthen the heart of the positive effect of muscle strength, improve cardiac output and enhance the perfusion of peripheral tissue, to compensate for the lack of oxygen caused by ischemia. However, the positive inotropic effect of persistent hyperexcitability of β_1_-AR leads to increased consumption of myocardial oxygen and relative shortage of energy supply to myocytes, which will induce myocardial organic hypertrophy, myocardial apoptosis, damage to the heart etc [26–28]. In the setting of heart failure, β_1_-AR with long-term stimulation by circulating catecholamine could lead to the down-regulation of β_1_-AR expression [29]. The down-regulation of β_1_-AR will reduce myocardial sensitivity to catecholamines, and attenuate positive effects of myocardium. It reduces myocardial energy consumption for the energy hungry state in heart failure, and results in saving the viability of the cells and resisting the excessive sympathetic nervous excitement caused by myocardial injury. It is helpful to slow its further deterioration and progression of heart failure. The internalization and desensitization of receptors were the mechanisms for the decrease of β_1_-AR in the cell membrane. Long-term excessive stimulation of β_1_-AR could induce the phosphorylation of the receptor, which initiates the process of receptor internalization [30, 31].

Receptors by endocytosis, from the cell membrane into the cell, can bind with the β-arrestins, which are degradated by the lysosomal, the other part can be returned to the cell membrane [32]. This internalization process leads to a decrease in the number of receptors. In addition, with sustained stimulation of catecholamines,β_1_-AR stayed activated and the sensitivity of β_1_-AR to catecholamines decreased. The biological function of β_1_-AR was reduced or lost, which is manifested by desensitization of the receptor to catecholamines. The number of non intrinsic receptors might be associated with the decrease of mRNA and the degradation of receptor molecules by cAMP dependent β_1_-AR [33]. The stability of mRNA was also decreased by increasing the RNA binding proteins [34]. β_1_-AR belongs to G protein-coupled-receptors that its desensitization is mainly divided into G proteins decoupled work and mRNA transcription or translation inhibition [35, 36]. However, the precise mechanism of β_1_-AR down-regulation in CIHF remains unclear.

Heart failure involves multiple pathophysiological processes such as interstitial fibrosis, cardiomyocyte apoptosis, and inflammatory response etc. In the present study, we found that let-7a, b, c, d, f and I were significantly upregulated and let-7e downregulated in rat failure hearts. This discrepancy of let-7 family expression may be attributed to the family members’ transcript from different gene clusters which contribute to different processes in heart failure. Nuclear GATA4 is a critical transcription factor for cardiac development and a variety of cardiac pathophysiological processes, and can be activated by phosphorylation through the cAMP-PKA signaling pathway [37]. Phosphorylated GATA4 not only had a wide range of regulatory effects on the expression of genes but also in the regulation of miRNAs expression [38–40]. GATA4 and its phosphorylated form are up-regulated by β_1_-AR/cAMP/PKA signaling pathway in CIHF [41].

Negative feedback regulation is one of the crucial physiological mechanisms and has been demonstrated to mediate a wide range of biological processes [42]. miRNAs are a class of negative regulator by acting on the 3’UTR (3’-untranslated region) of their target mRNAs, resulting in the formation of negative regulation feedback. Accumulative evidence has demonstrated the feedback regulation between miRNAs and their target genes in physiological and pathological processes. For instance, miR-326 inhibits Notch expression and is also negatively regulated by Notch, establishing a regulatory feedback loop [43]. Transcription factor E2F3 is identified as a direct target of miR-200b that is negatively regulated by E2F3b, forming a double-negative feedback loop between E2F3b and miR-200b [44, 45]. Recently Pulikkan *et al*. demonstrated that miR-223 targets and inhibits E2F1 which binds to the miR-223 promoter in AML blast cells and inhibits miR-223 transcription, generating negative feedback loop between these two molecules [46].

We employed the decoy ODN and siRNA assays to inhibit GATA4, and used DOB and FSK to stimulate cAMP-PKA signaling pathway to activate GATA4. Our results revealed that GATA4 positively regulated let-7a expression that in turn reduced β_1_-AR expression by acting on the 3’UTR of ADRB1 mRNA. It may be therefore conceived that there is a negative feedback loop which regulates β_1_-AR expression in CIHF. This negative feedback pathway is described as that β_1_-AR is excited and up-regulated in early phase of myocardial ischemia, which activates transcription factor GATA4 via cAMP/PKA signaling pathway, leading to up-regulation of let-7a which inhibits β_1_-AR expression in CIHF. Thus, it forms a critical feedback loop signaling pathway of β_1_-AR/cAMP/PKA/GATA-4/let-7/β_1_-AR, and the decreasing of β_1_-AR in CIHF is induced by β_1_-AR activation in early phase of CIHF, which is a kind of self-regulation of β_1_-AR in the course of ischemia induced heart failure. The limitations of the study may be that (1) there is a lack of transgenic overexpression or knockout model to further confirm the function of let-7a;(2) the role of let-7a in human heart failure has not been explored.

This signaling pathway of β_1_-AR negative feedback loop which had been opened out as our work for the first time. It has a significant influence on the research of cardiac pathophysiology. It could reduce the damage of myocardium caused by β_1_-AR activation, and protect cardiac function. It was helpful to further cognize the pathological process of CIHF with the dynamic changes of β_1_-AR expression by means of clarifying the negative feedback loop. Artificial mediation on this feedback circuit by interfering let-7 expression may be a potential strategy and new idea for the regulation of β_1_-AR expression in the prevention and treatment of heart failure.

## MATERIALS AND METHODS

### Animals

Healthy adult male Sprague-Dawley (SD) rats (200 ± 20 g; Vitalriver, Beijing, China) used in the present study were cared under the standard animal room conditions (temperature at 21 ± 1°C, humidity of 55 ± 5%) with daily food and water for 1 week prior to experiments. All experimental procedures were in accordance with and approved by the institutional Animal Care and Use Committees of Harbin Medical University.

### Rat model of heart failure

All rats were anesthetized with intraperitoneal injection (i.p.) of ketamine (60 mg/kg) and xylazine (6 mg/kg). Then, tracheal cannula was performed with a polyethylene tube and ventilated with the TOPO small animal Ventilato (Kent, OH, USA), and then the chest was opened through the third intercostal space and propped ribs by a rib spreader. The pericardium was opened carefully to expose the heart. The left anterior descending coronary artery (LAD) was ligated using a 5/0 silk thread to create infarction of the LV free wall for 4 week to establish a rat model of chronic heart failure. After operation, rats were given penicillin (10^5^ units/day, im) for 7 days. After surgery, the rats were given food and water ad libitum.

### Primary cardiomyocytes isolation and primary cardiomyocytes isolation and culture from neonatal rats

Neonatal rat ventricular cells (NRVCs) were isolated from 1 to 3-day-old SD rats. The neonatal rats were sterilized with 75% ethanol before decapitated. The hearts were obtained and rapidly removed into DMEM (Hyclone Laboratories, Utah, USA). The isolated hearts were cut into l~3 mm pieces. The tissues were dissociated in 0.25% trypsin at 37°C and cell suspension was collected. After centrifugation (500 g, 5 min), the isolated cells were resuspended in DMEM containing 10% fetal bovine serum (Biological Industries, 04-001-1A), 100 units/ml penicillin and 100 μg/ml streptomycin. Then the cells were cultured in DMEM at 37°C in humidified air with 5% CO_2_ for 2h. After fibroblast adherence, the non-adherent cardiomyocytes were uniformly replanted into a 6-well plate at a density of 10^6^ per well, and incubated at 37°C in humidified air with 5% CO_2_ and 95% air.

### Synthesis of miRNA and its inhibitor

Let-7a mimics (sequence: 5’-UGAGGUAGUAGG UUGUAUAGUU-3’) and its antisense inhibitor AMO-let-7a (sequence: 5’-AACUAUACAACCUC CUACCUCA-3’) were synthesized by RiboBio (RiboBio, Guangzhou, China). Additionally, a scrambled sequence was used as negative control (NC) (sequence: 5’-UUCUCCGAACGUGUCACGU-3’) that was designed according to the Blast search of the human/rat genomic. The X-treme GENE siRNA Transfection Reagent (Roche) was used to perform for transfection. After transfection with let-7a mimics (100 nM) or AMO-let-7a (200 nM) for 36 h, the NRVCs were used for qRT-PCR and Western blot analysis.

### Western blot analysis

Total protein was extracted with RIPA Lysis buffer (50 mM TRIS-HCl pH 7.4, 150 mM NaCl, 1% NP40, and 0.25% Na-deoxycholate) mixed with 1% protease inhibitor cocktail (Roche Molecular Biochemicals, Basel, Switzerland) and admixing with 5X loading buffer (Beyitime) at 100°C for 5 min. The protein concentration was determined by BCA kit (Beyotime, Shanghai, China) according to the instruction. For Western blot analysis, denatured proteins(120 μg from NRVCs and 80 μg from tissues) were separated in 10% SDS-polyacrylamide gels and blotted to nitrocellulose membranes. Prior to incubating with primary antibody, the membranes were blocked with 5% skimmed milk in PBS for 2 h in room temperature. The membranes were incubated with diluted antibody in PBS at 4°C overnight. The membranes were washed with PBS-T (PBS containing 0.5% Tween 20), followed by re-probing with the fluorescence conjugated secondary antibody at room temperature for 1 h. Membranes were washed again with PBS-T prior to detection on Odyssey infrared scanning system (LI-COR Biosciences, Lincoln, USA). The Western blot bands were quantified using Odyssey 3.0 software and normalized to loading control. The antibody resources and dilution used are as follows: The antibody against β_1_-AR was obtained from Santa Cruz (Santa Cruz Biotechnology Inc., Santa Cruz, CA, USA). The antibodies against GATA-4 total and GATA-4 phosphate S262 were obtained from Abcam (Abcam, Cambridge, MA, UK). The antibody against GAPDH was obtained from Jinshan (Shanghai, China). The fluorescence conjugated secondary antibodies (LI-COR Bioscience, Lincoln, NE, USA).

### Quantitative reverse transcription-PCR (qRT-PCR)

Total RNA was extracted from cultured cells and tissues using a Trizol standard protocol (Invitrogen, Carlsbad, USA). The integrity, quantity, and purity of RNA were examined using Nano-Drop 3000 Spectrophotometer (Thermo Scientific, Wilmington, USA). For each sample, 500 ng of total RNA was converted to cDNA using High Capacity cDNA Reverse Transcripition Kit (Applied Biosystems, Foster City, USA). The relative expression levels of mRNAs and miRNAs were quantified by the mirVana qRT-PCR miRNA Detection Kit in conjunction with real-time RT-PCR with SYBR Green I (Applied Biosystems). The threshold cycle (Ct) was determined and relative mRNA and miRNA levels were calculated based on the Ct values and normalized to GAPDH or U6 level for each sample. The sequences of primers used in our qRT-PCR experiments are shown in Table 1.

**Table 1:** Primers Used in qRT-PCR Experiments

Gene name	primer
let-7a	Forward: 5’-GGGTGAGGTAGTAGGTTGTATTG-3’
	Reward: 5’-TGTCGTGGAGTCGGCAATTG-3
let-7b	Forward: 5’-GGTGAGGTGAGGTAGTAGGTTGT-3’
	Reward: 5’-TGTCGTGGAGTCGGCAATTG-3’
let-7c	Forward: 5’-GGTGAGGTAGTAGGTTGTATGG-3’
	Reward: 5’-TGTCGTGGAGTCGGCAATTG-3’
let-7d	Forward: 5’-GGGAGAGGTAGTAGGTTGCA-3’
	Reward: 5’-TGTCGTGGAGTCGGCAATTG-3’
let-7e	Forward: 5’-GGTGAGGTAGGAGGTTGTATAG-3’
	Reward: 5’-TGTCGTGGAGTCGGCAATTG-3’
let-7f	Forward: 5’-GGTGAGGTGAGGTAGTAGATTGT-3’
	Reward: 5’-TGTCGTGGAGTCGGCAATTG-3’
let-7i	Forward: 5’-GGTGAGGTAGTAGTTTGTGCTG-3’
	Reward: 5’-TGTCGTGGAGTCGGCAATTG-3’
U6	Forward: 5’-GCTTCGGCAGCACATATACTAAAAT-3’
	Reward: 5’-CGCTTCACGAATTTGCGTGTCAT-3’
β_1_-AR mRNA (product size: 150 bp)	Forward: 5’-AGCGCCGATCTGGTCATG-3’
	Reward: 5’-GACACACAGGGTCTCGATGCT-3’
GAPDH (product size: 203bp)	Forward: 5’-AAGAAGGTGGTGAAGCAGGC-3’
	Reward: 5’-TCCACCACCCAGTTGCTGTA-3’

### Preparation and transfection of decoy ODNs and siRNA

The sequence (5’-TGTGTCTGATAAATCAGAGATAACCCCACC-3’) of decoy oligonucleotides fragment for GATA-4 (GATA ODN), a scrambled oligonucleotides fragment (5’-TAAATTGGCCAAGTGTAGCTCCG TTTGTGA-3’) as a negative control (control ODN), GAGA-4 siRNA (sense: 5’-CGGAAGCCCA AGAAUCUGA-3’, antisense: 5’-UCAGAUUCU UGGGCU UCCG-3’) and its negative control (sense: 5’-UUCUCCGAA CGUGUCACGU-3’, antisense: 5’-ACGUGACACGUUCGGAGAA-3’) were synthesized by Invitrogen (Carlsbad, CA, USA). The ODNs (100 nM) and siRNAs (100 nM) were transfected into NRVCs with the X-treme GENE siRNA Transfection Reagent (Roche) for 48h.

### Construction of plasmid carrying the 3’UTR of β_1_-adrenergic receptor (ADRB1) gene and luciferase assay

Targetscan predicts the presence of a putative binding site for let-7 in the 3’UTR of ADRB1 mRNA, the gene encoding β_1_-AR, which is highly conserved among mammals. A segment containing the let-7 miRNA binding sites flanked by the Hand III and Sac I restriction sites and a scramble sequence as a negative control (NC) were synthesized by Invitrogen. The sequences were inserted separately into the pMD18T-simple vector (Invitrogen), and then transferred into the pMIR-REPORT^TM^ Luciferase miRNA Expression Reporter Vector (Ambion, Austin, TX, USA). pRLRenilla Luciferase Reporter vector (pRL-TK, Promega, Madison, WI, USA) was used as an internal control. The plasmids and miRNAs were co-transfected into HEK293 cells using X-treme GENE siRNA transfection reagent (Roche, Mannheim, Germany). The dual luciferase reporter assay kit (Promega) and the GloMax biological fluorescent tester were used to detect luciferase activity 36 h after transfection. The results were expressed as fold changes by normalizing the scaled data. The sequences of human and rat ADRB13’UTR containing the binding site for let-7a inserted into the vectors were as follows: The human β_1_-AR 3’UTR (position 704-711 of human ADRB13’UTR, 160 base pairs): 5’-CGAGCTCTTAAGCTCTTCTTGGAACAAGCCCCACCTTGCTTTCCTTGTGTAGGGCAAACCCGCTGTCCCCCGCGCGCCTGGGTGGTCAGGCTGAGGGATTTCTACCTCACCTGTGCATTTGCACAGCAGATAGAAAGACTTGTTTATATTAAGCTTGGG-3’. The rat β_1_-AR 3’UTR (position 712-719 of rat ADRB13’UTR, 139 base pairs):5’-CGAGCTCCTCGGTGGTCCTGCTGTGGGTCCTCTACCTCACTCTGTGCATATTGCACAGCAAGATAGAAAGACTTGTTTATATTAAACAGCTTATTTATGTATCAATATTAGTTGGAAGGACCAGGCGCTGAAGCTTGGG-3’.

### Construction and infection of lentivirus carrying pre-let-7a

Lentivirus vectors expressing mature let-7a, anti-miRNA-oligo of let-7a (AMO-let-7a) or NC sequence were constructed (Invitrogen, China). The heart of rats was exposed by performing the thoractomy in the third left intercostal space. Virus-containing solution (20μl, 10^8^TU) including pre-let-7a, pre-AMO-let-7a and pre-NC were injected into the cavity of the left ventricle of rat heart with ascending aortic artery clamped. After surgery, the rats were given food and water ad libitum and received penicillin (10^5^ Units/day, im) for 7 days.

### Echocardiography

Cardiac function was evaluated by the Vevo 2100 High-Resolution Imaging system (Visual Sonics, Toronto, ON, Canada) 4 weeks after LAD ligation and infection with virus-containing solution. Rats were positioned on a Rat Pad (part of the VisualSonicsVevo Integrated Rail System II) with an integrated heater. Body temperature of rats was maintained at 37°C. The M-mode tracings were recorded in both parasternal long and short axis views. Ventricular parameters including diastolic anterior wall thicknesses, diastolic posterior wall thicknesses and left ventricular systolic internal diameters were measured. Fractional shortening (FS) and ejection fraction (EF) were calculated automatically.

### Data analysis

All data are presented as mean ± SD. Statistical analysis was performed using Student's non-paired t test or One-way analysis of variance (ANOVA) followed by Bonferroni multiple comparison test. A value of *P* < 0.05 was considered statistically significant.
